# Investigation of infant deaths associated with critical congenital heart diseases; 2018–2021, Türkiye

**DOI:** 10.1186/s12889-024-17966-4

**Published:** 2024-02-12

**Authors:** Nilgün Çaylan, Sıddika Songül Yalçin, Başak Tezel, Oben Üner, Şirin Aydin, Fatih Kara

**Affiliations:** 1 Child and Adolescent Health Department, Ministry of Health, General Directorate of Public Health, Ankara, Turkey; 2 Faculty of Medicine, Department of Pediatrics, Division of Social Pediatrics, Hacettepe University, Ankara, 06100 Turkey; 3 Faculty of Medicine, Department of Public Health, Selçuk University, Konya, Turkey

**Keywords:** Critical congenital heart disease, Mortality, Infant death

## Abstract

**Background:**

The aim of this study was to examine the characteristics of infant mortality associated with critical congenital heart disease (CCHD).

**Methods:**

In a cross-sectional study, data for the study were obtained through Death Notification System, Birth Notification System and Turkish Statistical Institute birth statistics.

**Results:**

Of all infant deaths, 9.8% (4083) were associated with CCHD, and the infant mortality rate specific to CCHD was 8.8 per 10,000 live births. CCHD-related infant deaths accounted for 8.0% of all neonatal deaths, while the CCHD specific neonatal death rate was 4.6 per 10,000 live births. Of the deaths 21.7% occurred in the early neonatal, 30.3% in the late neonatal and 48.0% in the post neonatal period. Group 1 diseases accounted for 59.1% (*n* = 2415) of CCHD related infant deaths, 40.5% (*n* = 1652) were in Group 2 and 0.4% (*n* = 16) were in the unspecified group. Hypoplastic left heart syndrome was the most common CCHD among infant deaths (*n* = 1012; 24.8%). The highest CCHD related mortality rate was found in infants with preterm birth and low birth-weight while multiparity, maternal age ≥ 35 years, twin/triplet pregnancy, male gender, maternal education in secondary school and below, and cesarean delivery were also associated with higher CCHD related infant mortality rate. There was at least one non-cardiac congenital anomaly/genetic disorder in 26.1% of all cases.

**Conclusion:**

CCHD holds a significant role in neonatal and infant mortality in Türkiye. To mitigate CCHD-related mortality rates, it is crucial to enhance prenatal diagnosis rates and promote widespread screening for neonatal CCHD.

## Background

Congenital heart disease (CHD) is described as malformation of the heart or great vessels and is the most common group among congenital malformations. They occur in approximately 8–12 of every 1000 live births [1–6]. Critical congenital heart disease (CCHD) is used to describe cardiac lesions, some of which are duct-dependent, that require intervention and/or surgical treatment early in life. It is estimated that approximately 20–25% of all CHDs are in this group [7, 8].

Deaths from CHDs are declining worldwide, but remain high especially in developing countries in Africa and Asia [9, 10]. In a study evaluating CHD-related mortality in all age groups between 1959 and 2009 in England and Wales, while infants under 1 year old accounted for more than 60% of all CHD-related deaths in the years 1959–1963, this rate decreased to 22% in the years 2004–2008 [11]. In the study evaluating infant deaths between 2007 and 2012 in Turkey, CHD is the 4th leading cause of infant mortality [12]. According to the Ministry of Health (MoH) Infant Mortality 2012–2018 report, congenital anomalies rank second with 25% among all causes of infant death and CHD is the most common anomaly [13].

CCHDs consist of the diseases with high mortality. However, in recent years, the prognosis of CCHD has improved significantly due to advances in cardiac catheterization in newborns, advances in surgical and anesthesia techniques, and increased standards of care in intensive care units [9, 11]. Although such advances improve survival in infants with CCHD, the mortality rate remains relatively high [8, 10, 14–18]. Although there have been few local studies conducted in Turkey that reported high mortality rates for CCHD [19–22], there is currently no comprehensive national-level study evaluating CCHD mortality and related conditions. The aim of this study was to examine the prevalence and characteristics of infant mortality attributed to CCHD and to provide evidence for future preventive strategies. By identifying the factors that contribute to CCHD related mortality, healthcare professionals and policymakers can better understand the challenges and areas that require attention in providing care for infants with CCHD. The findings from this study could potentially contribute to the improvement of preventive strategies aimed at reducing mortality rates associated with CCHD.

## Methods

The study was planned as a retrospective cohort study from 1 January 2018 to 31 December 2021, using national data. Data were obtained through the following national data collection systems: Death Notification System (DNS), Birth Notification System (BNS), and Turkish Statistical Institute (TSI) birth statistics.

In Turkey, DNS was implemented in 2013 [23]. All infant deaths are registered in the system without limitation of gestational age and birth weight, and examined in detail by the “Provincial Infant Mortality Monitoring Committees” and their preventability is determined. BNS was created in order to record and monitor all births that occurred with the help of health personnel inside and outside the health institutions and that are reported verbally are recorded in this system [24]. In addition, the number of births according to years and some sociodemographic characteristics were obtained from TSI and used in rate calculations [25].

### Diagnostic codes

Among the International Statistical Classification of Diseases and Related Health Problems 10th Revision (ICD-10) diagnostic codes, Q20-Q28 codes are used to identify CHDs [26]. There are various definitions of CCHD in the literature, and the diseases included in the studies also vary. In the Neonatal CCHD Screening Guide published by the MoH of Türkiye, CCHDs were examined under two sub-headings as (a) primary target and (b) secondary target diseases, according to their probability of detection by newborn screening with pulse oximeter test [27]. In this study, primary target diseases were grouped under Group 1 and secondary target diseases were grouped under Group 2. Group 1 diseases and their ICD 10 codes are as follows: Tetralogy of Fallot (Q21.3), hypoplastic left heart syndrome (Q23.4), transposition of great arteries (Q20.3), total anomalous pulmonary venous return (Q26.2), pulmonary atresia with intact ventricular septum (Q22.0), truncus arteriosus (Q20.0), tricuspid atresia (Q22.4). Group 2 diseases and their ICD 10 codes are as follows: Aortic arch anomalies (Aortic coarctation, interrupted aortic arch, aortic atresia/hypoplasia; Q25.1, Q25.2, Q25.4), atrioventricular septal defect (Q21.2), Pulmonary valve stenosis (Q22.1), Single ventricle physiology diseases (double outlet right ventricle, double outlet left ventricle, double inlet left ventricle and hypoplastic right heart syndrome; Q20.1, Q20.2, Q20.4, Q22.6), Ebstein anomaly (Q22.5).

### Birth numbers

The number of live births for frequency calculations was obtained from TSI. The number of births by gender, maternal age at birth, maternal education, parity, number of fetuses, region, gestational age, birth weight, and delivery type were obtained from TSI and BNS.

### Study population

Mortality data were obtained from the DNS database. Among all infant deaths in the DNS between January 1st, 2018 and December 31st, 2021, cases were identified whose main or underlying cause of death contain at least one CCHD ICD-10 code. Since the study is based on a national database, it includes all mortality cases such as preoperative, intraoperative and postoperative periods, deaths at home or in the hospital etc. Apart from diagnoses, all other sections such as accompanying diseases and surgery information, autopsy results, and clinical explanation sections were also reviewed.

Cases with a confirmed diagnosis of CCHD were included in the study with the following data: Date of birth and death, gender, gestational age at birth (< 37; ≥37 weeks), birth weight (< 2500 g; ≥2500 g), maternal age at birth (< 20; 20–34; ≥35 years), mother and father education level (secondary school and below; high school and above), number of household members, number of follow-ups during pregnancy, history of consanguinity, history of miscarriage and/or stillbirth, maternal smoking status, type of pregnancy (normal, ART: assisted reproduction technique), number of pregnancies (nulliparity, multiparity), number of fetuses in pregnancy (single; multiple), delivery method (normal; cesarean section), province of residence, forensic examination and autopsy result, ICD-10 diagnostic codes, intervention/surgery information.

Birth weight by gestational age at birth is categorized as: SGA (small for gestational age, < 10th percentile), AGA (appropriate for gestational age, 10-90th percentile) and LGA (large for gestational age, < 90th percentile) [28]. Babies with a gestational week of < 37 at birth were considered preterm, and babies with a birth weight of < 2500gr were considered low birth weight (LBW). Infant deaths are categorized by time of death as: early neonatal (0–6 days); late neonatal (7–28 days); post- neonatal period (29–364 days).

Province of residence data was grouped according to the definition of five demographic regions in the Demographic and Health Surveys: West, South, Central, North and East [29].

### Statistical analysis

Data were analyzed using Microsoft Office Excel 2019 and IBM SPSS Statistics for Windows, Version 23.0 statistical software package. Arithmetic mean and standard deviation were used for continuous variables, and frequency and percentage distributions were used for categorical variables. The chi-square test was used when comparing the percentage distribution of categorical data between groups. If a significant difference (*p* < 0.05) was identified in the chi-square analysis for contingency tables with more than two rows or columns, specifically the 4 × 2, 3 × 2, and 2 × 3 design tables, additional analyses were performed to pinpoint the particular subgroups responsible for the observed differences.

We conducted multiple logistic regression analysis in Model 1 to examine potential independent variables influencing CCHD-related mortality in the mortality cohort. Variables included region, gender, number of fetuses during pregnancy, number of pregnancies, parity, maternal age, number of miscarriages/stillbirths, maternal education level, presence of chronic disease, smoking, and consanguineous marriage. In Model 2, CCHD type was added to the analysis. The adjusted odds ratio (AOR, adjusted estimated risk) and 95% confidence intervals (CI) were calculated. Type I error was pre-set at 0.05 for all analyses.

## Results

In this study, it was found that there was a total of 4,639,445 live births in the 2018 to 2021, and 41,490 of the live births resulted in infant death. In 2018–2021, the total neonatal mortality rate (NMR) was 57.5 per 10,000 live births, while the total infant mortality rate (IMR) was 89.4 per 10,000 live births (Table 1).

**Table 1:** Critical congenital heart disease related infant deaths among all infant mortality cases, Türkiye, 2018–2021

Years	Live birth n	Neonatal mortality, n	Infant mortality, n	Neonatal mortality rate (1/10,000)^&^	IMR (1/10,000)^&^	The proportion of neonatal mortality attributed to CCHD n (%)*	The proportion of infant mortality attributed to CCHD n (%)**	CCHD related NMR, (1/10,000) ^&^	CCHD related IMR,(1/10,000) ^&^
**2018**	1,255,258^r^	7440	11,517	59.3	91.8	553 (7.4)	1100 (9.6)	4.4	8.8
**2019**	1,188,524^r^	6813	10.693	57.3	90.0	552 (8.1)	1045 (9.8)	4.6	8.8
**2020**	1,115,821^r^	6080	9487	54.5	85.0	543 (8.9)	997 (10.5)	4.9	8.9
**2021**	1,079,842	6327	9793	58.6	90.7	474 (7.5)	941 (9.6)	4.4	8.7
**2018–2021**	4,639,445	26,660	41,490	57.5	89.4	2122 (8.0)	4083 (9.8)	4.6	8.8

^r^ Revised birth numbers published by Turkish Statistical Institute

IMR: Infant mortality rate; CCHD: Critical congenital heart disease; NMR: Neonatal mortality rate

* Ratio of CCHD-related neonatal deaths among all neonatal deaths in the relevant years

** Ratio of CCHD-related infant deaths among all infant deaths in the relevant years

^&^ 1/10,000 live birth

**Fig. 1:**
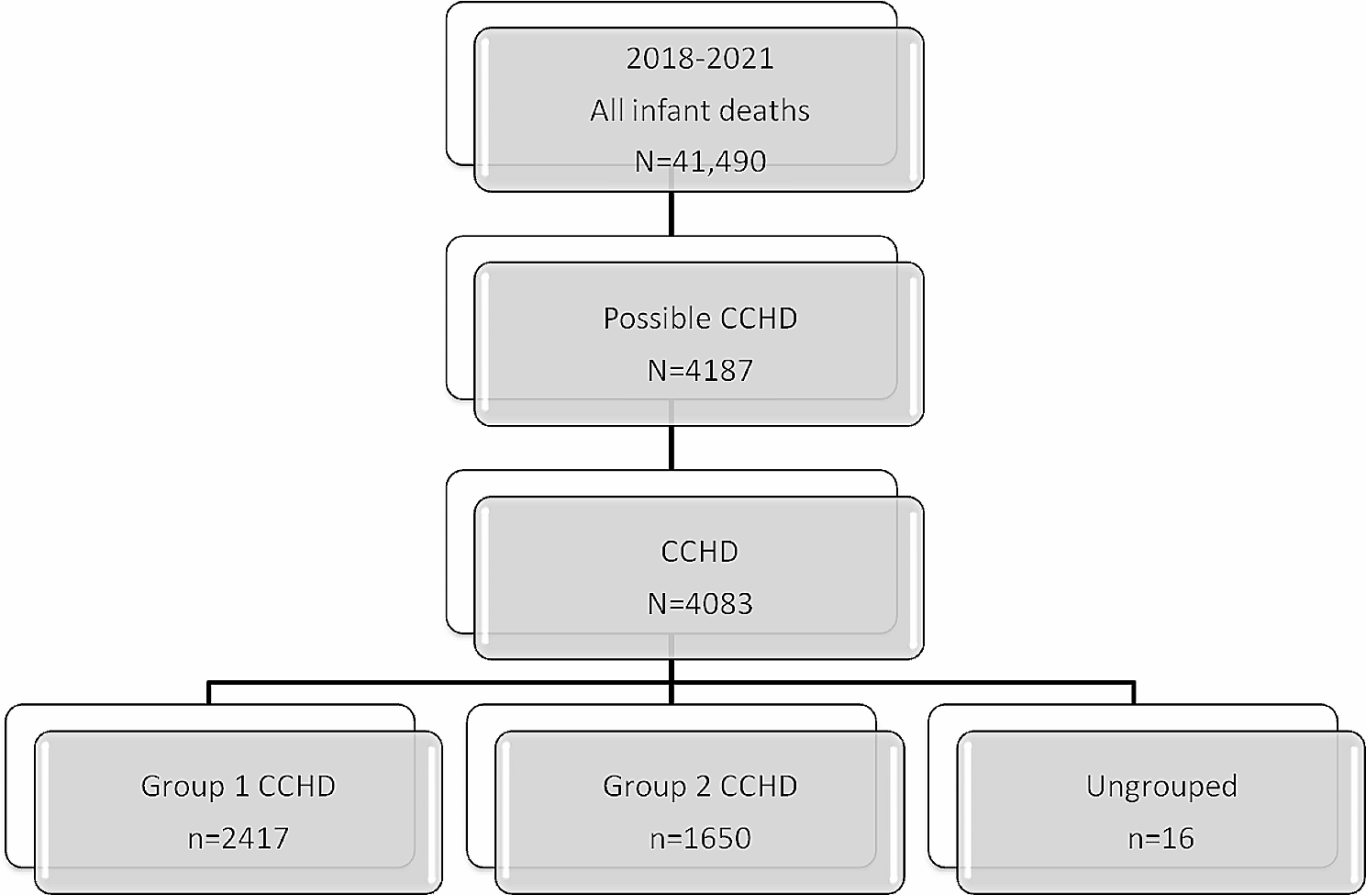
Flow chart of the study

By examining the causes of 41,490 infant deaths in four years (2018–2021), it was found that 4083 (9.8%) of infant deaths were associated with CCHD (Fig. 1). The infant mortality rate specific to CCHD was 8.8 per 10,000 live births. 52.0% of CCHD-related infant deaths occurred in the neonatal period (*n* = 2122/4083). CCHD related neonatal deaths accounted for 8.0% of all neonatal deaths, while the CCHD specific neonatal death rate was 4.6 per 10,000 live births (Table 1). While Group 1 diseases accounted for 59.2% (*n* = 2417 ) of CCHD related infant deaths, 40.4% (*n* = 1650 ) were in Group 2 and 0.4% (*n* = 16) were in the unspecified group. Hypoplastic left heart syndrome (*n* = 1012; 24.8%) is the most common CCHD among infant deaths, while atrioventricular septal defect (AVSD) (*n* = 637; 15.6%) 2nd and aortic arch anomalies (*n* = 603; 14.8%) ranked 3rd (Table 2).

**Table 2:** Distribution of CCHD related neonatal mortality rates and infant mortality rates by CCHD types, Türkiye, 2018–2021 (*n* = 4083)

CCHD type	CCHD related infant mortality n (%)^&^	CCHD related NMR (1/10,000)	CCHD related IMR (1/10,000)
**Group 1 diseases**	**2417 (59.2)**	**3.0**	**5.2**
Hypoplastic left heart syndrome	1012 (24.8)	1.5	2.2
Tetralogy of Fallot	461 (11.3)	0.4	1.0
Transposition of great arteries	422 (10.3)	0.4	0.9
Total anomalous pulmonary venous return	191 (4.7)	0.2	0.4
Pulmonary atresia with intact ventricular septum	149 (3.6)	0.2	0.3
Truncus arteriosus	125 (3.1)	0.1	0.3
Tricuspid atresia	57 (1.4)	0.06	0.1
***Group 2 diseases***	**1650 (40.4)**	**1.5**	**3.6**
Atrioventricular septal defect	637 (15.6)	0.3	1.4
Aortic arch anomalies*	603 (14.8)	0.7	1.3
Single ventricle physiology diseases**	272 (6.7)	0.3	0.6
Ebstein anomaly	73 (1.8)	0.1	0.2
Pulmonary valve stenosis	65 (1.6)	0.05	0.1
***Ungrouped***	**16 (0.4)**	**0.02**	**0.03**
**Total**	4083(100.0)	4.6	8.8

^**&**^Column percentage

*Aortic arch anomalies include aortic coarctation, interrupted aortic arch, aortic atresia/hypoplasia

^**^Single ventricle physiology diseases include double outlet right ventricle, double outlet left ventricle,

double inlet left ventricle, hypoplastic right heart syndrome.

NMR: Neonatal mortality rate; IMR: Infant mortality rate; CCHD: critical congenital heart disease

Table 3 shows the distribution of CCHD related infant mortality according to some socio-demographic variables and the comparison of Group 1 and Group 2 diseases related to CCHD in terms of these variables. The female/male ratio was 1/1.3. Of the infant deaths 21.7% occurred in the early neonatal, 30.3% in the late neonatal and 48.0% in the post neonatal period.

**Table 3:** Comparison of Group 1 and Group 2 CCHD-related infant deaths in terms of some variables, Türkiye, 2018–2021

Variables	All *n* = 4083 n (%)*	Group 1 *n* = 2417 %*	Group 2 *n* = 1650 %*	*p*
Gender				*< 0.001*
Female	1782 (43.6)	40.5	48.5	
Male	2301 (56.4)	59.5	51.5	
Age of mortality (day)				*< 0.001*
Early neonatal (0–6)	884 (21.7)	24.7^a^	17.2^b^	
Late neonatal (7–28)	1238 (30.3)	33.3^a^	26.1^b^	
Postneonatal (29–364)	1961 (48.0)	42.1^a^	56.7^b^	
Gestational age (w)				*< 0.001*
< 37	1235 (30.2)	27.7^a^	33.9^b^	
≥ 37	2846 (69.7)	72.3^a^	66.0^b^	
Unknown	2 (0.0)	0.0^a^	0.1^a^	
Birth weight (gr)				*< 0.001*
< 2500	1339 (32.8)	29.1^a^	38.3^b^	
≥ 2500	2742 (67.2)	70.9^a^	61.6^b^	
Unknown	2 (0.0)	0.0^a^	0.1^a^	
Number of fetuses in pregnancy				*0.965*
Singular	3852 (94.3)	94.4	94.3	
Twin/triplet	215 (5.3)	5.2	5.3	
Unknown	16 (0.4)	0.4	0.4	
Weight for gestational age				*0.002*
SGA	1241 (30.4)	28.6^a^	33.1^b^	
AGA	2620 (62.2)	66.3^a^	60.9^b^	
LGA	220 (5.4)	5.1^a^	5.9^a^	
Unknown	2 (0.0)	0.0^a^	0.1^a^	
Maternal age at birth (years)				*< 0.001*
< 20	189 (4.6)	4.9^a^	4.2^a^	
20–34	2946 (72.2)	76.6^a^	65.5^b^	
≥ 35	948 (23.2)	18.5^a^	30.3^b^	
Maternal education				*0.001*
Secondary school and below	2529 (61.9)	60.0^a^	64.8^b^	
High school and above	1453 (35.6)	37.8^a^	32.4^b^	
Unknown	101 (2.5)	2.2^a^	2.8^a^	
Paternal education				*0.010*
Secondary school and below	2161 (52.9)	51.5^a^	55.2^b^	
High school and above	1794 (43.9)	45.7^a^	41.2^b^	
Unknown	128 (3.1)	2.8^a^	3.6^a^	
Number of household members				*< 0.001*
≤ 4	2886 (70.7)	73.5^a^	66.4^b^	
> 4	1062 (26.0)	23.7^a^	29.6^b^	
Unknown	135 (3.3)	2.8^a^	4.0^a^	
Number of antenatal follow-up				*< 0.001*
< 8	1816 (44.5)	42.6^a^	47.2^b^	
≥ 8	2162 (53.0)	55.4^a^	49.5^b^	
Unknown	105 (2.6)	2.0^a^	3.4^b^	
Consanguineous marriage				*0.006*
Yes	915 (22.4)	21.6^a^	23.4^a^	
No	3120 (76.4)	77.6^a^	74.8^b^	
Unknown	48 (1.2)	0.8^a^	1.8^b^	
History of miscarriage/stillbirth				*0.002*
Yes	1233 (30.2)	28.7^a^	32.5^b^	
No	2802 (68.6)	70.4^a^	65.9^b^	
Unknown	48 (1.2)	0.9^a^	1.6^b^	
Parity				*0.002*
Nulliparity	1051 (25.7)	27.4^a^	23.2^b^	
Multiparity	2994 (73.3)	71.9^a^	75.5^b^	
Unknown	38 (0.9)	0.7^a^	1.3^b^	
Mode of pregnancy				*0.093*
Normal	3867 (94.7)	95.0	94.2	
ART	180 (4.4)	4.4	4.5	
Unknown	36 (0.9)	0.6	1.3	
Mode of delivery				*0.826*
Vaginal	1342 (32.9)	33.3	32.3	
Cesarean	2736 (67.0)	66.6	67.6	
Unknown	5 (0.1)	0.1	0.1	
Non-cardiac anomaly/genetic disorder				*< 0.001*
Yes	1067 (26.1)	16.4	40.5	
No	3016 (73.9)	83.6	59.5	
Referral to another hospital				*< 0.001*
Yes	1951(47.8)	50.1^a^	44.4^b^	
No	1970 (48.2)	47.1^a^	49.9^a^	
Unknown	162 (4.0)	2.8^a^	5.6^b^	

*Column percentage

^a,b^Different letters in the same line are statistically significant; ART: Assisted reproductive technology; SGA: Small for gestational age; AGA: Appropriate for gestational age; LGA: Large for gestational age; CCHD: critical congenital heart disease

The prevalence of preterm birth and LBW were 30.2% and 32.8% in all cases of CCHD related infant deaths. Of the cases, 30.4% had a history of SGA and 5.4% had a history of LGA birth. The prevalence of twin/triplet pregnancy and ART was 5.3% and 4.4%, respectively, and 73.3% of the infants were born from the pregnancy of multiparous mothers. In 30.2% of the cases, the mother had a history of miscarriage/stillbirth in her previous pregnancy. The majority of the mothers were between the ages of 20–34 (72.2%) and had secondary school or below education level (61.9%). While the number of people living in the household was 4 or less in 70.7% of the cases, the frequency of consanguineous marriage was 22.4%. The number of 8 or more antenatal follow-up visits in the current pregnancy was found in 53.0% of the cases. It has been reported that 67.0% of the babies were born by cesarean section and 47.8% of them were referred from one hospital to another hospital in the process that resulted in death (Table 3).

Male gender was more common in Group 1 disease compared to Group 2 (59.5% vs. 51.5%; *p* < 0.001). In Group 1, 42.1% of mortality occurred in the postneonatal period, while it was 56.7% in Group 2 (*p* < 0.001). The prevalence of preterm birth and LBW was higher in Group 2 compared to Group 1 (*p* < 0.001). In Group 1 compared to Group 2, the rate of both mother and father having high school and above education was higher (*p* = 0.001; *p* = 0.010, respectively). The rate of living 4 or less people in the household (73.5% vs. 66.4%) and 8 or more the antenatal follow-up visit rate (55.4% vs. 49.5%) was higher in Group 1 compared to Group 2 (*p* < 0.001). A history of miscarriage/stillbirth in previous pregnancies was higher in Group 2 compared to Group 1 (32.5% vs. 28.7%; *p* = 0.002). Nulliparity rate was higher in Group 1 compared to Group 2 (27.4% vs. 23.2%; *p* = 0.002). Infants with Group 1 disease were more likely to be referred to another hospital compared to Group 2 during the mortality process (50.1% vs. 44.4%; *p* < 0.001) (Table 3). There was no significant difference between Group 1 and Group 2 in terms of consanguineous marriage, mode of pregnancy, number of fetuses in pregnancy, and mode of delivery (Table 3).

Table 4 shows the differences in IMR associated with CCHD by socio-demographic characteristics. Male gender, 35 years and older maternal age at birth, secondary school and below maternal education level, multiparity, twin/triplet pregnancy, preterm birth, LBW, and cesarean delivery were associated with higher CCHD-related-IMR. The highest CCHD-related-IMR was found in infants born with LBW (37.2 per 10,000 live births) and preterm (24.5 per 10,000 live births). When examining regional differences, the lowest CCHD specific mortality rate was observed in the West (8.4 per 10,000), while the highest mortality rates were observed in the Central, Southern and Eastern regions (9.1; 9.0; 9.0 per 10,000, respectively) (Table 4).

**Table 4:** Infant mortality rates specific to CCHD by some sociodemographic characteristics, Türkiye, 2018–2021

Variables	Live birth n	Infant mortality n	CCHD related infant mortality n	IMR*	CCHD related IMR*
**Gender**					
Male	2,380,005	22,697	2301	95.4^a^	9.7^a^
Female	2,259,440	18,783	1782	83.1^b^	7.9^b^
**Maternal age at birth (years)**					
< 20	199,991	2551	189	127.6^a^	9.5^a^
20–34	3,657,162	30,725	2946	84.0^b^	8.1^b^
≥ 35	737,521	8089	948	109.7^c^	12.9^c^
**Maternal education**					
Secondary school and below	2,328,059	25,659	2529	110.2^a^	10.9^a^
High school and above	2,182,849	13,570	1453	62.2^b^	6.7^b^
**Parity**					
Nulliparity	1,679,472	11,803	1051	70.3^a^	6.3^a^
Multiparity	2,915,233	28,116	2994	96.4^b^	10.3^b^
**Number of fetuses in pregnancy**					
Singular	4,469,509	34,558	3852	77.3^a^	8.6^a^
Twin/triplet	143,729	5747	215	399.8^b^	15.0^b^
Gestational age (w)**					
≥ 37	1,879,157	6989	1333	37.2^a^	7.1^a^
< 37	246,561	12,269	605	497.6^b^	24.5^b^
Birth weight (gr)**					
≥ 2500	1,954,493	7089	1302	36.3^a^	6.7^a^
< 2500	171,117	12,116	636	708.1^b^	37.2^b^
Mode of delivery **					
Normal	831,886	5279	612	63.5^a^	7.4^a^
Cesarean	1,294,598	13,053	1325	100.8^b^	10.2^b^
**Regions**					
West	1,683,392	11,975	1417	71.1^a^	8.4
South	590,105	5090	533	86.3^b^	9.0
Central	840,496	6562	766	78.1^c^	9.1
North	234,738	1664	201	70.9^a^	8.6
East	1,290,714	16,199	1166	125.5^d^	9.0

*1/10,000 live birth; **For these variables, 2020–2021 live birth numbers in the Birth Notification System and 2020-2021 infant mortality numbers in Death Notification System were used

^a,b,c,d^ Different letters in the same column for variables are statistically significant (*p* < 0.05)

CCHD: critical congenital heart disease; IMR: Infant mortality rate

In cases of CCHD related infant death, diagnoses were also evaluated in terms of other non-cardiac anomalies/genetic disorders (Table 5). There was at least one non-cardiac congenital anomaly or genetic disorder in 26.1% of all cases (*n* = 1067 cases). Chromosomal anomalies, digestive system and musculoskeletal system anomalies were the most common associated congenital anomaly/genetic disorders. In cases of atrioventricular septal defect, an additional anomaly was present in 65.3%, while in Tetralogy of Fallot cases, it was observed in 36.2%. Single ventricle physiology diseases exhibited an accompanying anomaly in 29.4%, and a quarter of cases with pulmonary valve stenosis and aortic arch anomalies had an additional anomaly. In more than half of atrioventricular septal defect cases, a chromosomal anomaly diagnosis was confirmed. Tetralogy of Fallot cases reported a chromosomal anomaly in 11.3% and a digestive system anomaly in 8.9%.

**Table 5:** The frequencies of associated anomalies according to critical congenital heart diseases

	Total cases	Associated anomalies		Chromosomal anomalies		Digestive system		Musculo-skeletal system		Nervous system			Cleft plate/lip		Respiratory system		Urogenital system		Multiple anomalies	
	N	n	%	n	%	n	%	n	%		n	%	n	%	n	%	n	%	n	%
Atrioventricular septal defect**	637	416	65.3	342	53.7	21	3.3	19	3.0		12	1.9	13	2.0	3	0.5	7	1.1	18	2.8
Tetralogy of Fallot*	461	167	36.2	52	11.3	41	8.9	15	3.3		10	2.2	9	2.0	12	2.6	9	2.0	25	5.4
Single ventricle physiology diseases**	272	80	29.4	23	8.5	11	4.0	7	2.6		9	3.3	12	4.4	7	2.6	4	1.5	9	3.3
Pulmonary valve stenosis**	65	17	26.2	3	4.6	3	4.6	2	3.1		2	3.1	2	3.1	0	0.0	0	0.0	3	4.6
Aortic arch anomalies**	603	148	24.5	41	6.8	19	3.2	26	4.3		15	2.5	8	1.3	14	2.3	12	2.0	26	4.3
Tricuspid atresia*	57	10	17.5	0	0.0	4	7.0	2	3.5		0	0.0	0	0.0	3	5.3	1	1.8	0	0.0
Truncus arteriosus*	125	21	16.8	3	2.4	5	4.0	3	2.4		1	0.8	3	2.4	1	0.8	2	1.6	6	4.8
Total anomalous pulmonary venous return*	191	30	15.7	5	2.6	9	4.7	4	2.1		2	1.0	4	2.1	4	2.1	2	1.0	0	0.0
Pulmonary atresia with intact ventricular septum*	149	22	14.8	4	2.7	5	3.4	4	2.7		1	0.7	1	0.7	4	2.7	0	0.0	3	2.0
Hypoplastic left heart syndrome*	1012	124	12.3	26	2.6	27	2.7	25	2.5		16	1.6	14	1.4	9	0.9	6	0.6	12	1.2
Ebstein anomaly**	73	8	11.0	0	0.0	2	2.7	0	0.0		3	4.1	2	2.7	1	1.4	0	0.0	0	0.0
Transposition of great arteries*	422	22	5.2	3	0.7	8	1.9	2	0.5		0	0.0	1	0.2	1	0.2	1	0.2	3	0.7
Ungrouped***	16	2	12.5	0	0.0	1	6.3	0	0.0		1	6.3	0	0.0	0	0.0	0	0.0	0	0.0
Total	4083	1067	26.1	502	12.3	156	3.8	109	2.7		72	1.8	69	1.7	59	1.4	44	1.1	105	2.6

*Group 1 diseases; **Group 2 diseases; *** Group 3 diseases; %: row percentages

In the analysis of mortality within CCHD groups, logistic regression was employed to assess the risk of non-cardiac anomalies/genetic disorders based on regional, maternal, and infant characteristics. The findings revealed a lower risk of non-cardiac anomalies in CCHD mortality cases in the East and South regions compared to the Western region (*p* = 0.010, *p* = 0.005; respectively). Maternal age also played a significant role, with those aged ≥ 35 years having a 2.72 times higher risk of non-cardiac congenital anomalies (95% CI: 2.30–3.22) compared to those aged 20–34 years. Additionally, female infants exhibited a 1.38 times higher risk of non-cardiac anomalies (95% CI: 1.19–1.60) compared to male infants (*p* < 0.001). In Model 2, Group 2 CCHD cases showed a 3.25 times higher risk for associated anomalies than Group 1 diseases (Table 6).

**Table 6:** Possibility of accompanying non-cardiac anomaly in critical congenital heart diseases according to mother-infant characteristics*

	Model 1			Model 2		
	AOR	95% CI	*P*	AOR	95% CI	*P*
Region						
West	1.00			1.00		
South	0.72	0.57–0.93	*0.010*	0.68	0.52–0.87	*0.003*
Central	0.91	0.74–1.12	*0.362*	0.86	0.70–1.07	*0.171*
North	0.87	0.61–1.22	*0.416*	0.76	0.53–1.09	*0.130*
East	0.76	0.62–0.92	*0.005*	0.72	0.59–0.88	*0.001*
Maternal age at birth (years)						
< 20	1.21	0.83–1.74	*0.321*	1.24	0.85–1.82	*0.264*
20–34	1.00			1.00		
≥ 35	2.72	2.30–3.22	*< 0.001*	2.39	2.01–2.85	*< 0.001*
Maternal education						
Middle school and below	1.03	0.88–1.21	*0.702*	0.98	0.83–1.15	*0.775*
High school and above	1.00			1.00		
Maternal chronic disease						
No	1.00			1.00		
Yes	0.99	0.79–1.24	*0.918*	1.00	0.79–1.26	*0.965*
Maternal smoking						
No						
Yes	1.07	0.81–1.41	*0.646*	1.04	0.78–1.38	*0.806*
Consanguineous marriage						
No	1.00			1.00		
Yes	1.01	0.85–1.22	*0.877*	0.97	0.80–1.17	*0.733*
Parity						
Nullipar	1.00			1.00		
Multipar	1.00	0.82–1.22	*0.985*	1.01	0.83–1.24	*0.906*
History of abortion/stillbirth						
No	1.00			1.00		
Yes	1.10	0.92–1.30	*0.293*	1.07	0.90–1.28	*0.444*
Number of fetuses in pregnancy						
Singular	1.00			1.00		
Twin/triplet	0.80	0.57–1.12	*0.188*	0.77	0.54–1.09	*0.143*
Gender						
Male	1.00			1.00		
Female	1.38	1.19–1.60	*< 0.001*	1.28	1.09–1.49	*0.002*
Critical congenital heart disease						
Group 1 diseases				1.00		
Group 2 diseases				3.25	2.79–3.79	*< 0.001*
Constant	0.26		*< 0.001*	0.17		*< 0.001*

Logistic regression analysis was performed. CI: Confidence interval, AOR: Adjusted odds ratio

## Discussion

In the current study, mortality of CCHD was analyzed using national data. It was shown that the CCHD-specific NMR was 4.6 per 10,000 live births, and CCHD-specific-IMR was 8.8. In our study, CCHD-related neonatal deaths accounted for 8.0% of all newborn deaths, and CCHD-related infant death cases accounted for 9.8% of all infant deaths for the years 2018–2021. We also evaluated the changes in CCHD related IMR over the years. In 2020, it is noteworthy that the rate of CCHD-related neonatal and infant deaths increased along with the decrease in both the overall neonatal death rate and infant mortality rate. It is thought that this difference in rates in 2020 may be related to the measures implemented due to the COVID-19 pandemic (lock down and mask use etc.) indirectly reducing the spread of other respiratory tract agents. However, this issue needs to be investigated in order to confirm the cause of these changes in infant mortality.

The mortality and survival of CCHD varies in various countries and regions around the world [8, 10, 14–17, 19, 20, 30]. In a study conducted in Brazil between 2014 and 2016 evaluating survival and risk factors for death in newborns with critical and/or complex congenital heart disease in the neonatal period, the death rate was found to be 8.1 per 10,000 live births [14]. In a study in which the results of fifteen congenital anomaly monitoring programs in Europe, North and South America and Asia were analyzed together, it was reported that there were significant differences between countries in terms of mortality in the first month of life [15]. In the study, while the neonatal mortality rate was found to be highest in Argentina (25.5%) and Malta (24.1%), the lowest neonatal mortality was found in Italy-Emilia Romagna (4.0%), Germany-Saxony Anhalt (5.4%). In Norway between 2014 and 2016, the mortality rate was 10% in 2359 live-born babies with severe CHD. 58% of them died before surgery and 81% of preoperative deaths were during palliative care [30]. In a study in which 105 patients with CCHD who were followed up in a tertiary neonatal intensive care unit in Türkiye between 2010 and 2012, the mortality rate was 35.2% while in another tertiary center in 2017–2018, evaluating perioperative mortality in cases requiring intervention in the neonatal period, the overall mortality and the intervention mortality were found 27% and 22% respectively [19, 20]. Several factors linked to CCHD mortality have been documented in prior studies. The literature suggests that a low rate of prenatal diagnosis, hindrances to the termination of pregnancy (ToP), and delayed postnatal diagnoses may contribute to high mortality rates [14, 15, 22]. Similar challenges are believed to contribute to the high CCHD mortality observed in Türkiye. Prenatal and postnatal early diagnosis rates in the country still fall short of the desired levels [19, 22, 31, 32]. Although the MoH has issued recommendations and provided guidelines for neonatal pulse oximetry screening before hospital discharge, the procedure is not obligatory [27, 31, 33]. In contrast, some countries have successfully integrated pulse oximetry screening into routine neonatal care [34]. It is noteworthy that while there are no legal barriers to implementing ToP for medical reasons in Türkiye, some families opt to decline this option, even in cases involving anomalies incompatible with life, for various reasons [35, 36]. Addressing these issues through enhanced prenatal and postnatal screening measures and potentially reconsidering the mandatory nature of newborn CCHD screening could contribute to reducing CCHD-related mortality in Türkiye.

In previous studies, the type of CCHD, comorbidities, accompanying congenital anomalies, preterm birth, LBW, multiple pregnancy are among the factors associated with high mortality rates [10, 14, 17–20, 30]. In a Norwegian study, it was reported that comorbidity and univentricular CHDs are common among infants resulting in mortality [30]. Similarly, in our study, the most common disease among mortality cases was hypoplastic left heart syndrome. In our study, mortality data were also analyzed in terms of accompanying non-CCHD anomalies/genetic disorders. There was at least one congenital anomaly/genetic disorder other than CCHD in 26.1% of the mortality cases. The most common genetic disorders/congenital anomalies were chromosomal abnormalities, digestive system and musculoskeletal anomalies. Likewise, a recent multicenter study conducted in Turkey revealed a 21.4% incidence of extracardiac malformations in cases (*n* = 98) associated with CCHD mortality [22].

In our study, preterm birth, LBW, twin/triplet pregnancy, multiparity, old maternal age, low maternal educational level, cesarean birth and male gender were determined as the conditions associated with higher CCHD mortality rates. In addition, the referral rate of 47.8% in the mortality process is one of the important clues that may be associated with the high mortality rates, and the referral rate in Group 1 patients is higher than in Group 2 diseases.

In our study, we also investigated the regional differences in CCHD mortality. There were regional differences in infant mortality rates. However, no statistically significant regional difference was found in death rates specific to CCHD. In a recent study, it was observed that the Eastern region had a lower incidence of CCHD cases, and the lifespan of children with CCHD was shorter [37]. In the current study, although there were regional variations in overall infant mortality rates, no statistically significant differences were found in death rates specifically related to CCHD. This suggests the possibility of divergent diagnostic rates for CCHD cases among different regions. The Eastern region, characterized by higher home births, higher birth rates, lower maternal education, and lower socioeconomic status [29], prompts the need for field studies to thoroughly investigate this paradoxical situation.

### Limitations and strengths of the study

The study has some limitations. All the independent variables examined were limited to the available data in the database. For this reason, some important independent variables that may be associated with mortality were not included in the study (prenatal diagnosis, postnatal screening, time of diagnosis, and frequency of late diagnosis, mothers' underlying disease, relationship between mortality and surgery etc.). CCHD diagnoses obtained from the database are directly related to the knowledge and awareness of healthcare professionals and the use of appropriate disease-specific ICD-10 codes. Recently, in order to reduce the rate of under-reporting of causes of death, data entries are audited by medical inspectors assigned by the MoH and counseling service is provided regarding coding. Another limitation was that only infant deaths were included in this study. The real extent of mortality could be learned by determining the rate of CCHD in stillbirths and ToP cases.

On the other hand, there are some strengths of this study. Our study includes 4-year national infant mortality data. It is a nationally representative study since the entire universe is examined.

## Conclusions

This study highlights the considerable impact of CCHD related infant mortality rates, underscoring its significance as a prominent cause of neonatal and infant deaths. To address and mitigate these high mortality rates, it is crucial to emphasize the importance of enhancing prenatal diagnosis rates and widespread adoption of neonatal CCHD screening, as recommended by the MoH. Additionally, further in-depth investigations are warranted to identify and understand additional factors influencing the survival of individuals with CCHD. The establishment of a comprehensive birth defect surveillance system is highly recommended. Such a system would not only aid in identifying potential preventive strategies but also allow for the monitoring of intervention outcomes to enhance overall healthcare efforts in this critical area.

## Data Availability

The data that support the findings of this study are available from the Turkish Ministry of Health but restrictions apply to the availability of these data, which were used under license for the current study, and so are not publicly available. Data are however available from the corresponding author upon reasonable request and with permission of Ministry of Health.
